# Three-dimensional condylar and glenoid fossa changes following functional appliance therapy in class II malocclusion: a systematic review

**DOI:** 10.3389/fdmed.2026.1870531

**Published:** 2026-06-17

**Authors:** Sunidhi Kamboj, Nishi Joshi, Akhil Shetty

**Affiliations:** 1 Department of Orthodontics and Dentofacial Orthopedics, AB Shetty Memorial Institute of Dental Sciences, NITTE (Deemed to be University), Mangalore, India; 2 Department of Preventive and Pediatric Dentistry, AB Shetty Memorial Institute of Dental Sciences, NITTE (Deemed to be University), Mangalore, India

**Keywords:** CBCT, class II malocclusion, condyle, functional appliance, glenoid fossa, mandibular growth, temporomandibular joint, three-dimensional imaging

## Abstract

**Background:**

Functional appliance therapy is widely used for the management of Class II malocclusion in growing patients, aiming to enhance mandibular growth through anterior repositioning of the mandible. This forward positioning is believed to induce adaptive remodeling within the temporomandibular joint (TMJ), particularly in the condyle and glenoid fossa. With the advent of cone-beam computed tomography (CBCT), three-dimensional evaluation of these structures has become more precise, however, existing evidence remains heterogeneous and inconclusive.

**Objective:**

To systematically evaluate three-dimensional changes in condylar morphology, position, and glenoid fossa remodeling following functional appliance therapy in growing patients with Class II malocclusion.

**Methods:**

This systematic review was conducted in accordance with PRISMA guidelines and registered in PROSPERO (CRD420261379151). Electronic databases including PubMed, Scopus, Web of Science, Cochrane Library, and Google Scholar were searched up to December 2025. Studies involving growing patients treated with functional appliances and assessed using CBCT were included. Data extraction and quality assessment were performed independently by two reviewers using the Newcastle–Ottawa Scale and RoB 2 tool. Due to heterogeneity, a narrative synthesis was undertaken.

**Results:**

Seven studies comprising 258 patients were included. Twin Block appliances demonstrated significant increases in condylar volume and forward positioning, whereas Herbst appliances showed modest increases in condylar dimensions with relatively stable condyle–fossa relationships. Glenoid fossa remodeling was inconsistently reported. Overall, TMJ changes were mild to moderate, with variability across studies.

**Conclusion:**

Functional appliances induce adaptive three-dimensional changes in the condyle, while glenoid fossa remodeling is limited and inconsistent. Skeletal correction in Class II malocclusion appears multifactorial, with TMJ adaptation contributing modestly to treatment outcomes.

## Introduction

Functional appliances are widely used in orthodontics for the correction of Class II malocclusion, particularly in growing patients with mandibular retrognathia. The term “functional appliance” encompasses a range of removable and fixed devices designed to influence the development, function, and spatial position of the mandible. These appliances act by repositioning the mandible in the anteroposterior and vertical planes, thereby inducing a combination of dentoalveolar and orthopedic changes that contribute to skeletal correction.

The fundamental principle of functional appliance therapy involves advancing the mandible and maintaining it in a protruded position for a sustained period, with the aim of enhancing mandibular growth. This forward positioning results in anterior displacement of the condyle within the glenoid fossa, which is believed to stimulate adaptive remodeling within the temporomandibular joint (TMJ). Evidence from human studies suggests that functional appliance therapy can lead to remodeling of the glenoid fossa, often characterized by anterior and inferior repositioning, along with a modest increase in mandibular length and posterior redirection of condylar growth (1). These structural changes are considered critical in achieving skeletal correction in Class II malocclusion.

Experimental animal studies have further demonstrated increased cellular proliferation and metabolic activity within the condylar cartilage following mandibular advancement with fixed functional appliances, supporting the biological plausibility of treatment-induced TMJ remodeling (2). However, the magnitude, direction, and clinical significance of these changes remain subjects of debate, particularly when comparing removable and fixed functional appliance modalities.

The advent of cone-beam computed tomography (CBCT) has significantly enhanced the ability to evaluate craniofacial structures with high spatial resolution. CBCT employs a cone-shaped x-ray beam and a rotating tube-detector system to acquire volumetric data, which are reconstructed into three-dimensional images using algorithms such as the modified Feldkamp algorithm (3). This imaging modality allows accurate visualization of the condyle, glenoid fossa, and joint spaces in multiple planes, overcoming the inherent limitations of conventional two-dimensional imaging techniques.

Recent studies utilizing CBCT have provided detailed insights into three-dimensional changes in condylar morphology, position, and glenoid fossa adaptation following functional appliance therapy (4, 5). These investigations have reported increases in condylar volume, alterations in joint space relationships, and remodeling of the glenoid fossa, suggesting that TMJ adaptation plays a significant role in treatment outcomes. Moreover, differences in the pattern and extent of remodeling have been observed between various functional appliances, particularly between removable appliances such as the Twin-block and fixed functional systems like the Herbst and Forsus appliances (6, 7).

Despite the growing body of evidence, the literature remains heterogeneous in terms of study design, imaging protocols, outcome measures, and appliance types. Existing systematic reviews have primarily focused on overall skeletal effects or individual appliances, with limited emphasis on comprehensive three-dimensional evaluation of TMJ remodeling (8, 9). Furthermore, direct comparisons between different functional appliances in terms of their impact on condylar and glenoid fossa changes are scarce.

Therefore, there is a need for a systematic review that synthesizes current evidence on three-dimensional condylar and glenoid fossa changes following functional appliance therapy in Class II malocclusion. Such an analysis would provide a clearer understanding of the biological mechanisms underlying treatment effects, identify potential differences between appliance types, and support evidence-based clinical decision-making in orthodontics.

## Methodology

**Figure 1:**
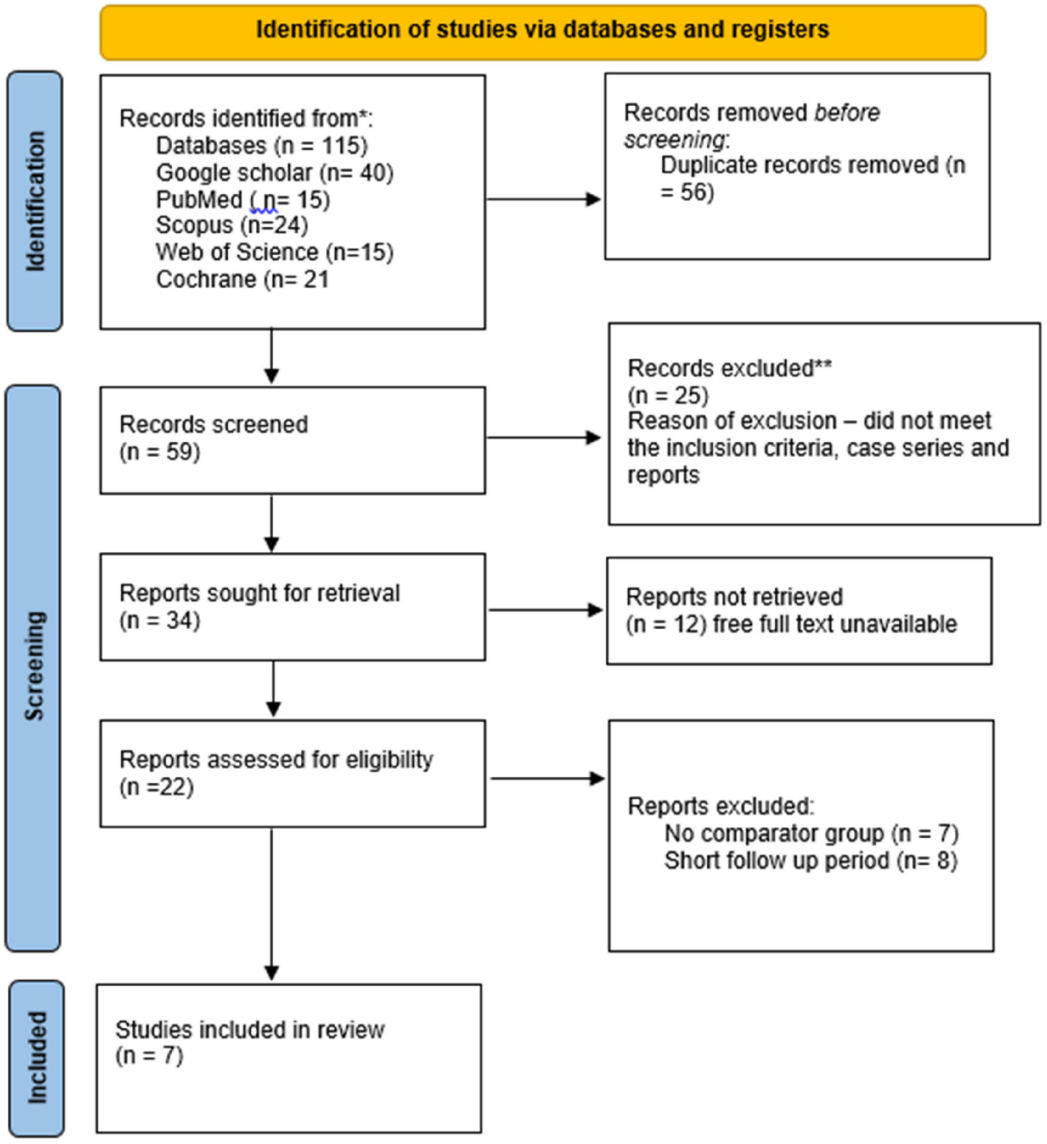
PRISMA flow diagram.

This systematic review was conducted in accordance with the PRISMA (Preferred Reporting Items for Systematic Reviews and Meta-Analyses) statement (10) and the guidelines outlined in the Cochrane Handbook for Systematic Reviews of Interventions (11). (Figure 1) PICO (Population, Intervention, Comparison, and Outcome) framework was employed to formulate a focused research question and to define the inclusion and exclusion criteria for the present study (12). The review protocol was registered in the PROSPERO database (CRD420261379151). Registration was completed after the initial screening phase but prior to final data extraction and synthesis.

## Inclusion and exclusion criteria for study selection

The inclusion criteria for selecting studies in the PICOS frame work was:

**Population (P):** Growing patients (children and adolescents) diagnosed with **Class II malocclusion** (skeletal and/or dental), with no prior orthodontic or orthopedic treatment done.

**Intervention (I):** Treatment using **functional appliances** for mandibular advancement, including twin block, Herbst appliance, forsus (FRD), MARA or another similar functional appliance.

**Comparison (C):** Untreated Class II control groups and/or Comparison between different functional appliances.

**Outcome(O):**

**Primary Outcomes**—Changes in Condylar volume, condylar height, glenoid fossa depth, temporomandibular joint (TMJ) joint spaces

**Secondary Outcomes**- Changes in condylar morphology, condylar positional changes, glenoid fossa remodeling, skeletal parameters

**Study Design (S):** Randomized controlled trials (RCTs), controlled clinical trials, prospective and retrospective cohort studies, CBCT-based observational studies

## Literature search

Multiple electronic databases, including Google Scholar, PubMed, Web of Science, Scopus, Cochrane Library were searched using a combination of keywords and Medical Subject Headings (MeSH) terms. The final electronic search was conducted in December 2025. In addition to electronic database searching, manual screening of reference lists of included studies and relevant review articles was performed to identify additional eligible studies. Duplicate records were removed using reference management software prior to screening (Table 1).

**Table 1:** Search strategy and search terms used.

DATABASE	SEARCH QUERY	RESULTS
Google scholar	"class II malocclusion” AND (“Twin Block” OR “Herbst appliance” OR “functional appliance”) AND (condyle OR “glenoid fossa” OR “temporomandibular joint”) AND (CBCT OR “cone beam computed tomography”)	40
PubMed	(“Class II malocclusion” OR “mandibular retrognathism”) AND (“Functional appliance” OR “Twin Block” OR “Herbst appliance” OR “fixed functional appliance” OR “Xbow”) AND (“Temporomandibular joint” OR “condyle” OR “glenoid fossa” OR “TMJ”) AND (“Cone-Beam Computed Tomography” OR “CBCT” OR “3D imaging”)	15
Scopus	TITLE-ABS-KEY (“class II malocclusion” OR “mandibular retrognathism”) AND TITLE-ABS-KEY (“twin block” OR “Herbst appliance” OR “functional appliance” OR “fixed functional appliance” OR “xbow”) AND TITLE-ABS-KEY (“condyle” OR “glenoid fossa” OR “temporomandibular joint” OR “TMJ”) AND TITLE-ABS-KEY (“CBCT” OR “cone beam computed tomography” OR “3D imaging”)	24
Cochrane	(“Class II malocclusion” OR “mandibular retrognathism”) AND (“Twin Block” OR “Herbst appliance” OR “functional appliance”) AND (“temporomandibular joint” OR “condyle” OR “glenoid fossa”)	21
Web of science	TS = (“class II malocclusion” OR “mandibular retrognathism”) AND TS = (“twin block” OR “Herbst appliance” OR “functional appliance” OR “fixed functional appliance” OR “xbow”) AND TS = (“condyle” OR “glenoid fossa” OR “temporomandibular joint”) AND TS = (“CBCT” OR “cone beam computed tomography” OR “3D imaging”)	15

The search strategy combined terms related to *Class II malocclusion, mandibular retrognathia, functional appliance, Twin Block, Herbst appliance, Forsus FRD, MARA, temporomandibular joint condyle, condylar remodeling, glenoid fossa, CBCT, and three-dimensional imaging*. Boolean operators (AND, OR) were used to combine the search terms effectively. No filters on date restrictions were placed, only English language publications were considered for inclusion.

### Eligibility criteria

**Inclusion criteria**

- Population: Growing patients (children and adolescents) diagnosed with Class II malocclusion, with no prior orthodontic or orthopedic treatment.
- Intervention: Treatment using functional appliances for mandibular advancement, including Twin-block, Herbst, Forsus (FRD), MARA, or similar appliances.
- Comparison: Studies including untreated Class II control groups and/or comparisons between different functional appliances.
- Outcomes: studies reporting at least one temporomandibular joint (TMJ)-related outcome, condylar morphology, condylar volume or surface area, Glenoid fossa depth or remodeling, TMJ joint spaces, condylar position changes
- Study designs: Randomized controlled trials, controlled clinical trials, prospective and retrospective cohort studies, and CBCT-based observational studies.
- Language: English language articles only.

**Exclusion criteria**

Studies meeting any of the following criteria were excluded:

- Case reports or case series with fewer than 10 participants
- Review articles, meta-analyses, editorials, or conference abstracts without full text
- Animal or *in vitro* studies
- Studies involving adult patients only or non-growing individuals
- Patients with craniofacial syndromes, cleft lip/palate, systemic conditions, or pre-existing TMJ disorders
- Studies not involving functional appliances or not evaluating mandibular advancement
- Studies reporting only dental or soft tissue outcomes without TMJ-specific data
- Studies lacking quantitative data or adequate outcome reporting
- Studies without imaging-based TMJ assessment

## Data extraction

The following information was collected and tabulated:

- Author and year
- Sample size
- Age group
- Sex
- Type of fixed appliance used
- Radiographic method
- Parameters assessed
- Follow up period
- Outcomes

Two reviewers (S.K and N.J.) independently screened titles and abstracts for eligibility. Full texts of potentially eligible articles were assessed independently. Disagreements were resolved through discussion, and where necessary, consultation with a third reviewer (A.S.). The detailed characteristics of each of the included studies is explained in Table 2. Due to the heterogeneity of the studies and lack of quantitative outcomes in the included studies, a narrative synthesis was done. Possible sources of heterogeneity among included studies were explored qualitatively through structured subgroup comparisons. Studies were grouped and compared based on age and sex, types of functional appliance used, parameters assessed, follow up period and the outcomes of every study.

**Table 2:** Characteristics of included articles.

Author & Year	Sample Size	Age Group	Sex	Study design	Type of Fixed Appliance Used	Radiographic Method	Parameters Assessed	Follow-up Period	Outcomes
Vilefort et al., 2019 (20)	76	8–16 yrs (pre- & pubertal groups)	Not specified clearly (mixed)	Retrospective case control	Herbst appliance	CBCT	Condyle–glenoid fossa positional changes and rotations	8–12 months	No significant change in condyle–fossa relationship; minimal displacement (<0.75 mm)
Jiang et al., 2020 (16)	26	Growing patients (CVM stage 3–4)	TB: 9M, 8F; Control: 4M, 5F	retrospective cohort study	Twin Block (removable functional)	CBCT	Condylar volume, surface area, joint spaces, condylar position	∼8 months	Significant increase in condylar size and positional changes; greater TMJ remodeling vs. control
Hassan et al., 2020 (17)	24	11–14 yrs (mean ∼12.7 yrs)	13F, 11M	Prospective single-arm clinical study	Twin Block (functional appliance)	CBCT	Condylar volume, length, width, height; angular (ANB, gonial, saddle); linear (Co-Gn, CoR-CoL, facial height)	∼7–9 months	increase in Condylar volume & mandibular dimensions; decrease ANB & facial convexity; significant skeletal changes
Nindra et al., 2021 (19)	30	12–16 yrs	7M, 8F per group	retrospective observational study	Herbst appliance (fixed functional); MBT fixed appliance + elastics	CBCT	Condylar height, volume, glenoid fossa remodeling	8–10 months (Herbst); ∼14–16 months (fixed)	Herbst showed significant ↑ in condylar height & volume and anterior GF remodeling vs. fixed appliance
Mohamed et al., 2020 (18)	24	9–12 yrs	12M, 12F	Prospective study	Twin Block (functional; removable, not fixed)	CBCT	Condylar volume, condylar position, skeletal parameters	9 months	Significant increase in condylar volume and forward positioning in both groups; LLLT had no additional effect
Friesen et al., 2025 (21)	54	10–16 yrs	Mixed (approx. equal)	3-arm parallel randomized clinical trial	Herbst & Xbow (fixed functional appliances)	CBCT	3D positional changes of condyle & glenoid fossa (AP, vertical, mediolateral)	∼12.4 months	No significant positional changes in condyle/fossa vs. control; minimal remodeling effects
Chávez-Sevillano et al., 2025 (25)	24	∼11.9–12.5 yrs	TB: 7M,5F; HB: 6M,6F	a randomized clinical trial	Herbst (fixed functional); Twin Block (removable)	CBCT	Condyle & glenoid fossa 3D displacement	12 months	Both TB & Herbst showed posterior and superior condylar growth; similar effects on condyle and GF

Differences in comparator technique were examined across these groups to identify consistent patterns and potential sources of variability. Meta-regression was not performed due to the absence of pooled quantitative data.

## Risk of bias assessment

**Figure 2:**
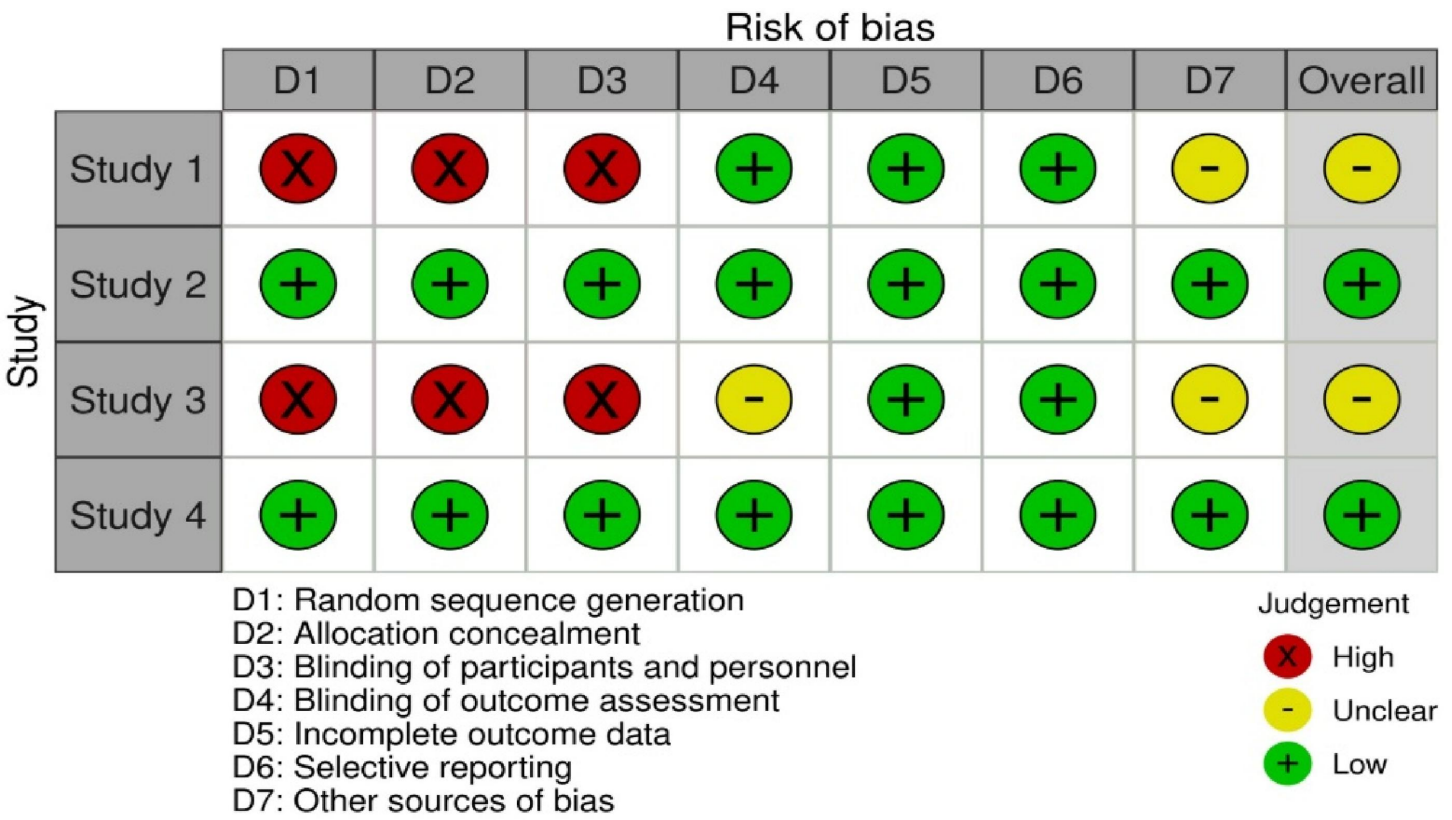
Quality of included studies assessed with Newcastle–Ottawa scale.

**Figure 3:**
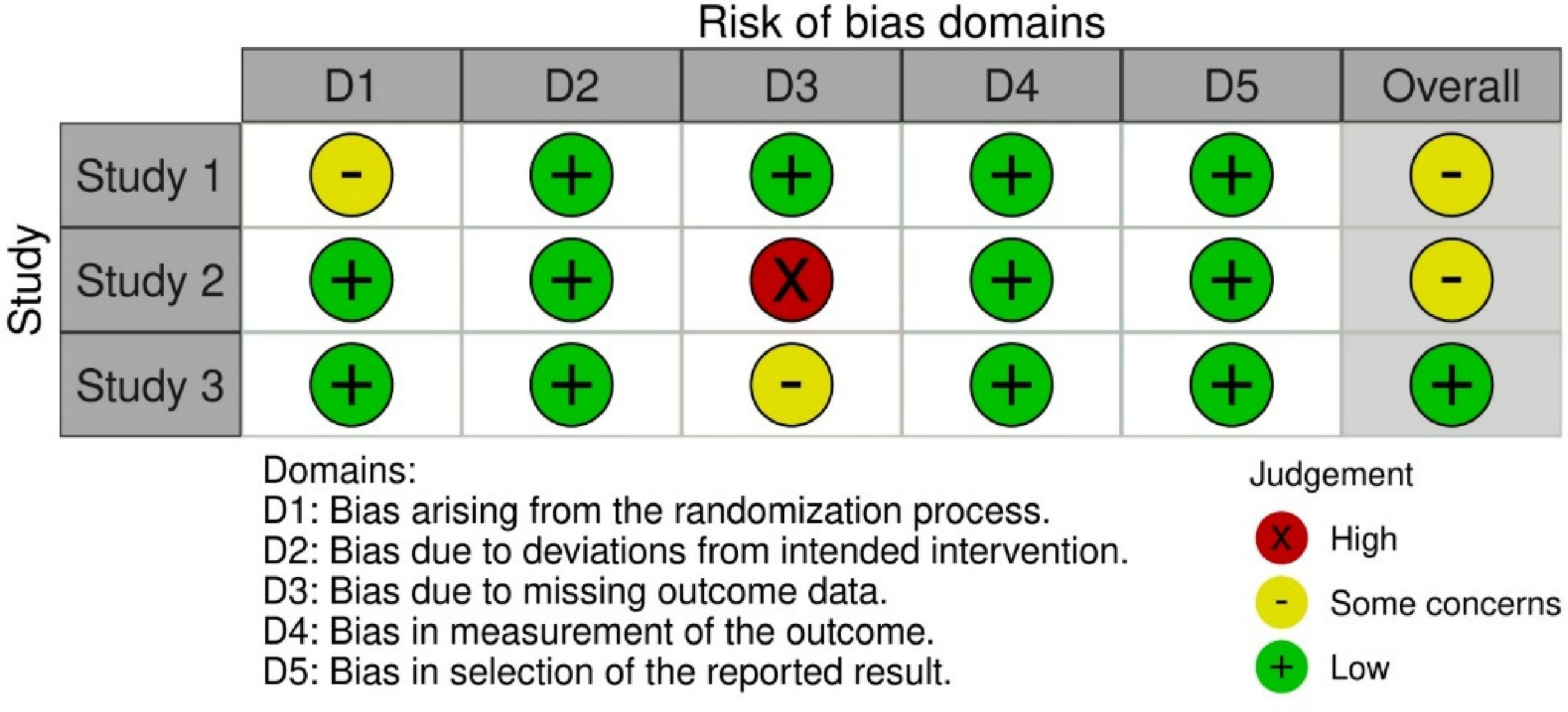
Quality of included studies assessed with RoB 2.

Risk of bias assessment was performed independently by two reviewers (S.K and N.J). Observational studies were assessed using the Modified Newcastle–Ottawa Scale (NOS) (13), whereas randomized clinical trials were evaluated using the Cochrane RoB 2 (14) tool (Table 3). Robvis tool (15) was used to generate traffic light plots for the same Disagreements were resolved through discussion and consultation with a third reviewer when necessary (Figures 2, 3).

**Table 3:** Studies included for risk of bias assessment.

Modified Newcastle–Ottawa Scale	RoB 2
Study 1—Vilefort et al., 2019 (20)	Study 1—Mohamed et al., 2020 (18)
Study 2—Jiang et al., 2020 (16)	Study 2—Friesen et al., 2025 (21)
Study 3—Hassan et al., 2020 (17)	Study 3—Chávez-Sevillano et al., 2025 (25)
Study 4—Nindra et al., 2021 (19)	

Certainty of evidence was considered using the GRADE (Grading of Recommendations Assessment, Development and Evaluation) framework. However, a formal GRADE assessment was not undertaken because of the substantial clinical and methodological heterogeneity among the included studies, including differences in study design, functional appliance type, CBCT acquisition protocols, outcome measures, and follow-up duration. Furthermore, the limited number of studies and the absence of quantitative synthesis precluded a meaningful certainty-of-evidence assessment.

## Results

A total of seven studies fulfilling the inclusion criteria were included in this systematic review, comprising 258 growing patients with Class II malocclusion. All studies employed cone-beam computed tomography (CBCT) for three-dimensional evaluation of temporomandibular joint (TMJ) structures. The included populations predominantly consisted of pre-adolescent and adolescent individuals in active growth phases, with reported ages ranging from 8 to 16 years. Both removable and fixed functional appliances were evaluated, primarily including Twin Block and Herbst appliances, with one study also assessing the Xbow system. The duration of follow-up varied across studies, ranging from approximately 7 months to 16 months.

Across the included studies, consistent findings were observed regarding condylar adaptation following functional appliance therapy. Studies evaluating Twin Block therapy (Jiang et al., 2020; Hassan et al., 2020; Mohamed et al., 2020) reported a significant increase in condylar volume, surface area, and linear dimensions, The magnitude of condylar adaptation reported across studies was generally modest to moderate, with volumetric increases ranging from approximately 111 mm^3^ to 214 mm^3^ and condylar height increases of approximately 1–1.5 mm following functional appliance therapy along with forward positioning of the condyle (16–18). In contrast, studies investigating fixed functional appliances such as the Herbst (Nindra et al., 2021) (19) demonstrated increases in condylar height and volume, accompanied by evidence of anterior glenoid fossa remodeling. However, not all studies reported significant positional changes; Vilefort et al. (2019) and Friesen et al. (2025) observed minimal or no statistically significant changes in condyle–fossa relationships, with displacement generally less than 0.75 mm (21, 22).

Regarding condylar position, removable appliance groups demonstrated a tendency toward anterior displacement, whereas fixed appliance studies reported relatively stable condyle–fossa relationships. Glenoid fossa remodeling was inconsistently reported, with some studies indicating adaptive anterior remodeling while others found negligible changes. Some included studies suggested greater condylar volumetric changes with Twin Block therapy, whereas Herbst studies tended to report relatively stable condyle–fossa relationships. However, direct comparisons were limited and the heterogeneity of included studies prevents definitive conclusions regarding appliance superiority. Overall, the findings indicate that functional appliance therapy results in mild-to-moderate adaptive remodeling of the condyle, with variable effects on glenoid fossa morphology and condylar position.

## Discussion

The present systematic review evaluated three-dimensional condylar and glenoid fossa changes following functional appliance therapy in growing patients with Class II malocclusion using CBCT imaging. The findings suggest that functional appliances induce adaptive remodeling within the temporomandibular joint; however, the magnitude and clinical significance of these changes remain variable and, in many cases, modest.

The present review focused specifically on CBCT-based three-dimensional TMJ evaluation, allowing more detailed assessment of condylar and glenoid fossa adaptation than conventional two-dimensional studies.

A consistent finding across several included studies was the increase in condylar volume and dimensions, particularly in patients treated with Twin Block appliances. This observation is in agreement with previous CBCT-based investigations by Liu et al. (2020), who demonstrated significant increases in condylar volume and joint space changes following Twin Block therapy (4). Similarly, a systematic review by Perinetti et al. (2019) concluded that functional appliances are associated with measurable changes in condylar morphology, although the clinical relevance of these changes remains uncertain (8). The observed increase in condylar dimensions may be attributed to enhanced endochondral ossification and cellular proliferation within the condylar cartilage, as previously demonstrated in experimental studies (Pancherz, 1985) (6).

Despite these findings, not all studies included in the present review reported significant condylar remodeling. Vilefort et al. (2019) (20) and Friesen et al. (2025) (21) found minimal or no significant positional or morphological changes in the condyle or glenoid fossa. These discrepancies may be explained by differences in study design, treatment duration, patient age, and growth potential. Importantly, the role of natural growth cannot be excluded, as highlighted by Björk and Skieller, who emphasized that mandibular growth patterns are highly individualized and may overshadow treatment effects (22).

Another important consideration is the distinction between true condylar growth and positional adaptation. Studies involving Twin Block appliances frequently reported anterior displacement of the condyle, which may represent functional repositioning rather than permanent structural change. In contrast, Herbst appliance studies generally demonstrated stable condyle–fossa relationships, supporting earlier findings by Pancherz and Ruf (2000) (23), who reported that Herbst therapy results primarily in dentoalveolar changes with limited skeletal displacement of the TMJ structures. This suggests that the orthopedic effects of functional appliances may not necessarily involve significant repositioning of the condyle within the glenoid fossa.

The evidence regarding glenoid fossa remodeling remains inconclusive. While Nindra et al. (2021) (19) reported anterior remodeling of the glenoid fossa following Herbst therapy, other studies such as those by Friesen et al. (2025) (21) and Vilefort et al. (2019) (20) did not observe significant changes. These findings are consistent with previous literature indicating that glenoid fossa adaptation is less predictable and occurs to a lesser extent compared to condylar remodeling (Katsavrias, 2006) (24). It is possible that fossa remodeling requires longer observation periods or occurs at a rate that is not easily detectable within the relatively short follow-up durations of most clinical studies.

When comparing removable and fixed functional appliances, the present review suggests that Twin Block appliances may produce greater increases in condylar volume and more pronounced positional changes, likely due to intermittent mandibular advancement and greater neuromuscular adaptation. On the other hand, fixed functional appliances such as the Herbst provide continuous mandibular advancement, resulting in more stable but less pronounced skeletal effects. However, direct comparisons, such as those reported by Chávez-Sevillano et al. (2025), indicate that both appliance types may ultimately produce similar overall effects on condylar growth and glenoid fossa adaptation (25). These findings are supported by previous comparative studies (Mahamad et al., 2012; Flores-Mir et al., 2007), which concluded that differences between functional appliances are often minimal when treatment is performed during the appropriate growth period (7, 26).

Overall, the findings of this review reinforce the concept that the effects of functional appliance therapy are multifactorial. While TMJ remodeling contributes to treatment outcomes, skeletal correction is also influenced by dentoalveolar changes, neuromuscular adaptation, and inherent growth potential. This aligns with the conclusions of McNamara (1981) who emphasized that functional appliances modify growth patterns rather than create entirely new growth (27).

## Strengths and limitations

This systematic review has important methodological strengths. It was conducted in accordance with PRISMA guidelines and registered in PROSPERO, ensuring transparency and reducing reporting bias. A comprehensive literature searches across multiple databases, supplemented by manual screening, minimized the risk of missing relevant studies. Inclusion was restricted to CBCT-based studies, enabling accurate three-dimensional assessment of condylar and glenoid fossa changes. Independent screening, duplicate data extraction, and structured risk-of-bias assessment using both the Newcastle–Ottawa Scale and RoB 2 tool further strengthened the reliability of the findings.

Despite these strengths, several limitations should be acknowledged. Considerable heterogeneity was present across included studies in terms of study design, appliance type, sample characteristics, CBCT protocols, outcome variables, and follow-up duration, which prevented quantitative synthesis and meta-analysis. Most studies had small sample sizes and relatively short observation periods, limiting the assessment of long-term stability of temporomandibular joint (TMJ) adaptations. Additionally, a large proportion of the evidence was derived from observational studies, increasing the risk of selection bias and confounding, and making it difficult to clearly separate treatment effects from normal growth-related changes in growing patients. Variability in CBCT acquisition settings, segmentation techniques, and measurement methods further limited comparability between studies. A formal GRADE assessment was not performed due to substantial heterogeneity among the included studies and the absence of a meta-analysis, limiting assessment of the overall certainty of evidence. These factors collectively reduce the overall certainty of evidence and highlight the need for future well-designed randomized controlled trials with standardized imaging protocols, uniform outcome measures, and long-term follow-up to better understand true TMJ remodeling following functional appliance therapy.

## Conclusion

Within the limitations of the available evidence, functional appliance therapy in growing patients with Class II malocclusion results in adaptive three-dimensional changes in the condyle and, to a lesser extent, the glenoid fossa. Current evidence suggests that functional appliance therapy may be associated with adaptive three-dimensional condylar changes; however, the certainty of evidence remains limited due to heterogeneity and methodological limitations of available studies

While increases in condylar volume and dimensions are commonly observed, positional changes are minimal and often not clinically significant. Glenoid fossa remodeling is inconsistent and less pronounced. Differences between removable and fixed functional appliances exist but are not definitive. Overall, TMJ adaptation contributes to treatment outcomes; however, skeletal correction is multifactorial and influenced by growth, dentoalveolar changes, and neuromuscular adaptation.

## Clinical implications and future scope

Functional appliances induce mild adaptive TMJ remodelling, which should be interpreted alongside dentoalveolar changes and normal growth. Twin Block may promote greater condylar growth in compliant patients, while fixed appliances like the Herbst offer more predictable results with less reliance on compliance. Overall, treatment success in Class II malocclusion depends more on case selection and timing during growth than appliance type.

Future research should include larger, well-designed RCTs with standardized CBCT protocols and long-term follow-up to assess stability. Advanced 3D and AI-based imaging, along with standardized comparative studies and evaluation of treatment timing, are needed to improve evidence quality and clinical decision-making.

## References

[1] Popowich K Nebbe B Major PW. Effect of herbst treatment on temporomandibular joint morphology: a systematic literature review. Am J Orthod Dentofacial Orthop. (2003) 123(4):388–94. 12695765 10.1067/mod.2003.89

[2] Machado GL. CBCT Imaging—a boon to orthodontics. Saudi Dent J. (2015) 27(1):12–21. 10.1016/j.sdentj.2014.08.004 25544810 PMC4273277

[3] Ghali S Katti G Shahbaz S Katti C. Cone beam computed tomography: a boon for maxillofacial imaging. J Indian Acad Oral Med Radiol. (2017) 29(1):30. 10.4103/jiaomr.JIAOMR_89_16

[4] Liu X Chen X Li J Zhou Y. Three-dimensional cone-beam computed tomography analysis of temporomandibular joint response to the twin-block functional appliance. Angle Orthod. (2020) 90(3):401–8. 10.4041/kjod.2020.50.2.86 PMC7093662 32257934

[5] Mohamed MAH Abu Taleb NS Mahmoud NF. Three-dimensional assessment of mandibular condylar volume and position after twin-block therapy. Dent J (Basel). (2020) 8(4):115. 10.3390/dj8040115 33050123 PMC7712278

[6] Pancherz H. The herbst appliance—its biologic effects and clinical use. Am J Orthod. (1985) 87(1):1–20. 10.1016/0002-9416(85)90169-1 3855346

[7] Mahamad IK Neela PK Mascarenhas R Husain A. A comparison of twin-block and forsus (FRD) functional appliances—a cephalometric study. Int J Orthod Milwaukee. (2012) 23(3):49–58. 23094559

[8] Perinetti G Primožič J Franchi L Contardo L. Treatment effects of functional appliances on the temporomandibular joint: a systematic review with meta-analysis. Prog Orthod. (2019) 20(1):20. 10.1186/s40510-019-0272-2 31111270 PMC6527728

[9] Alhammadi MS Halboub E Fayed MS Labib A El-Saaidi C. Global distribution of malocclusion traits: a systematic review. Dent Press J Orthod. (2018) 23(6):40.e1–40.e10. 10.1590/2177-6709.23.6.40.e1-10.onl PMC6340198 30672991

[10] Page MJ McKenzie JE Bossuyt PM Boutron I Hoffmann TC Mulrow CD. The PRISMA 2020 statement: an updated guideline for reporting systematic reviews. Br Med J. (2021) 372:n71. 10.1136/bmj.n71 33782057 PMC8005924

[11] Higgins JPT Green S, editors. Cochrane Handbook for Systematic Reviews of Interventions. Version 5.1.0. London: The Cochrane Collaboration (2011). Available online at: http://www.cochrane-handbook.org (Accessed December 22, 2025).

[12] Amir-Behghadami M Janati A. Population, intervention, comparison, outcomes and study (PICOS) design as a framework to formulate eligibility criteria in systematic reviews. Emerg Med J. (2020) 37(6):387. 10.1136/emermed-2020-209567 32253195

[13] Wells G Shea B O'Connell D Peterson J Welch V Losos M. The Newcastle–Ottawa Scale (NOS) for Assessing the Quality of Nonrandomised Studies in Meta-analyses. Ottawa: Ottawa Hospital Research Institute (2014).

[14] Sterne JAC Savović J Page MJ Elbers RG Blencowe NS Boutron I. Rob 2: a revised tool for assessing risk of bias in randomised trials. Br Med J. (2019) 366:l4898. 10.1136/bmj.l4898 31462531

[15] McGuinness LA Higgins JPT. Risk-of-bias visualization (ROBVIS): an R package and shiny web app for visualizing risk-of-bias assessments. Res Synth Methods. (2021) 12:55–61. 10.1002/jrsm.1411 32336025

[16] Jiang YY Sun L Wang H Zhao CY Zhang WB. Three-dimensional cone beam computed tomography analysis of temporomandibular joint response to the twin-block functional appliance. Korean J Orthod. (2020) 50(2):86–97. 10.4041/kjod.2020.50.2.86 32257934 PMC7093662

[17] Kumar S Hassan N Anjan R Kumar K Anand B Bhowmick D. A CBCT comparison of condylar modifications in patients treated with twin block appliance. Ann Rom Soc Cell Biol. (2021) 25(2):719–27.

[18] Hameed Mohamed MA Abdallah KF Hussein FA. Three-dimensional assessment of mandibular condylar volume and position subsequent to twin block functional therapy of skeletal class II malocclusion accompanied by low-level laser therapy. Dent J. (2020) 8(4):115. 10.3390/dj8040115 PMC7712278 33050123

[19] Nindra J Sidhu MS Kochhar AS Dabas A Valletta R Rongo R. Three-dimensional evaluation of condyle-glenoid fossa complex following treatment with herbst appliance. J Clin Med. (2021) 10(20):4730. 10.3390/jcm10204730 34682852 PMC8538158

[20] Cheib Vilefort PL Farah LO Gontijo HP Moro A Ruellas AC Cevidanes LH. Condyle-glenoid fossa relationship after herbst appliance treatment during two stages of craniofacial skeletal maturation: a retrospective study. Orthod Craniofac Res. (2019) 22(4):345–53. 10.1111/ocr.12338 31419375

[21] Friesen R Aldajani T Kaipatur NR Lai H Hernandez IA Major PW. Temporomandibular joint condylar/fossa positional changes after herbst and Xbow treatments in adolescents assessed through cone-beam computed tomography imaging: a randomized controlled trial. Am J Orthod Dentofacial Orthop. (2025) 167(5):515–25. 10.1016/j.ajodo.2025.01.016 40379387

[22] Björk A Skieller V. Growth of the maxilla in three dimensions as revealed radiographically by the implant method. Br J Orthod. (1977) 4(2):53–64. 10.1179/bjo.4.2.53 273440

[23] Ruf S Pancherz H. Does bite-jumping damage the TMJ? A prospective longitudinal clinical and MRI study of herbst patients. Angle Orthod. (2000) 70(3):183–99. 10.1043/0003-3219(2000)070<0183:DBJDTT>2.0.CO;2 10926428 10.1043/0003-3219(2000)070<0183:DBJDTT>2.0.CO;2

[24] Katsavrias EG. Morphology of the temporomandibular joint in subjects with class II division 2 malocclusions. Am J Orthod Dentofacial Orthop. (2006) 129(4):470–8. 10.1016/j.ajodo.2005.01.018 16627172

[25] Chávez-Sevillano MG Carvalho FD Miguel JA Batista KB Fernandes LQ Blanco-Victorio DJ. Three-dimensional condyle and glenoid fossa alterations after class II treatment with twin block and herbst functional appliances–a randomized clinical trial. Eur J Orthod. (2025) 47(4):cjaf038. 10.1093/ejo/cjaf038 40795311

[26] Flores-Mir C Ayeh A Goswani A Charkhandeh S. Skeletal and dental changes in class II division 1 malocclusions treated with splint-type herbst appliances. Angle Orthod. (2007) 77(2):376–81. 10.2319/0003-3219(2007)077[0376:SADCIC]2.0.CO;2 17319777 10.2319/0003-3219(2007)077[0376:SADCIC]2.0.CO;2

[27] McNamara JJ Jr. Components of class II malocclusion in children 8–10 years of age. Angle Orthod. (1981) 51(3):177–202. 10.1043/0003-3219(1981)051<0177:COCIMI>2.0.CO;2 7023290 10.1043/0003-3219(1981)051<0177:COCIMI>2.0.CO;2

